# Case report: Pediatric hepatopulmonary syndrome despite strict weight control after craniopharyngioma surgery

**DOI:** 10.3389/fendo.2024.1459451

**Published:** 2024-10-30

**Authors:** Satoko Yoshikawa, Tomozumi Takatani, Rieko Takatani, Ayano Inui, Tomoo Fujisawa, Hiromichi Hamada

**Affiliations:** ^1^ Department of Pediatrics, Graduate School of Medicine, Chiba University, Chiba, Japan; ^2^ Faculty of Education, Graduate School of Education, Chiba University, Chiba, Japan; ^3^ Department of Pediatric Hepatology and Gastroenterology, Saiseikai Yokohama-shi Tobu Hospital, Yokohama, Japan

**Keywords:** hepatopulmonary syndrome, nonalcoholic steatohepatitis, craniopharyngioma, growth hormone, obesity

## Abstract

Childhood-onset craniopharyngiomas, though rare, are intracranial malformations that can cause obesity by disrupting the hypothalamus, a condition that often persists even after tumor resection. This severe obesity increases the risk of diabetes and fatty liver disease in childhood. Concurrently, panhypopituitarism, including growth hormone (GH) deficiency, may develop. Notably, some individuals with GH deficiency may exhibit a normal growth rate, making GH therapy unnecessary for growth purposes. However, in these cases, GH therapy may still be beneficial in preventing the progression of nonalcoholic fatty liver disease or nonalcoholic steatohepatitis. Although weight management is traditionally considered the gold standard for preventing liver cirrhosis, its effectiveness can be limited by hypothalamic dysfunction and the difficulty of achieving successful weight control. Our case study highlights a patient with normal growth despite GH deficiency, who did not receive GH replacement therapy and continued to struggle with hypothalamic obesity. Despite effective body weight control, the patient developed hepatopulmonary syndrome, indicating that relying solely on weight management may not be sufficient to prevent liver complications. This case underscores the importance of addressing GH deficiency even when growth is normal. Our findings suggest that GH replacement therapy could be beneficial for preventing liver cirrhosis in such cases.

## 1 Introduction

Childhood-onset craniopharyngiomas are rare intracranial embryonal malformations of the sellar region (1). Some patients develop severe obesity, known as hypothalamic obesity, due to hypothalamic disruption, which can persist even after tumor resection (2–4). This severe obesity can lead to complications such as diabetes and nonalcoholic fatty liver disease (NAFLD) even in childhood. Although managing weight gain is reported to prevent such complications, it often fails because hypothalamic disruptions can increase appetite and decrease energy expenditure (5), potentially resulting in severe liver cirrhosis (6). Craniopharyngiomas can also cause deficiencies in one or more anterior pituitary hormones, including growth hormone (GH). Recent studies suggest that GH deficiency contributes to liver disease in patients with hypothalamic injury-induced obesity; however, GH replacement therapy has been shown to improve or prevent liver diseases (7, 8). Despite this, the effectiveness of GH replacement therapy, in conjunction with lifestyle modifications, remains unconfirmed. Many studies report that obesity and GH deficiency worsen in patients with hypothalamic disruption, often leading to the progression of fatty liver disease (6, 8, 9).

This case report describes a patient with hypothalamic obesity who subsequently developed severe liver disease, including hepatopulmonary syndrome (HPS). Despite having GH deficiency, his growth rate remained normal; therefore, GH replacement therapy was deemed unnecessary. He adhered to a strict diet, successfully managing his body weight and improving his body mass index standard deviation score (BMI-SDS). However, he still developed liver cirrhosis and poor oxygenation, indicative of HPS. This suggests that body weight reduction alone, which is typically the first-line treatment for NAFLD, is insufficient, and GH replacement therapy may be necessary.

## 2 Case description

**Figure 1:**
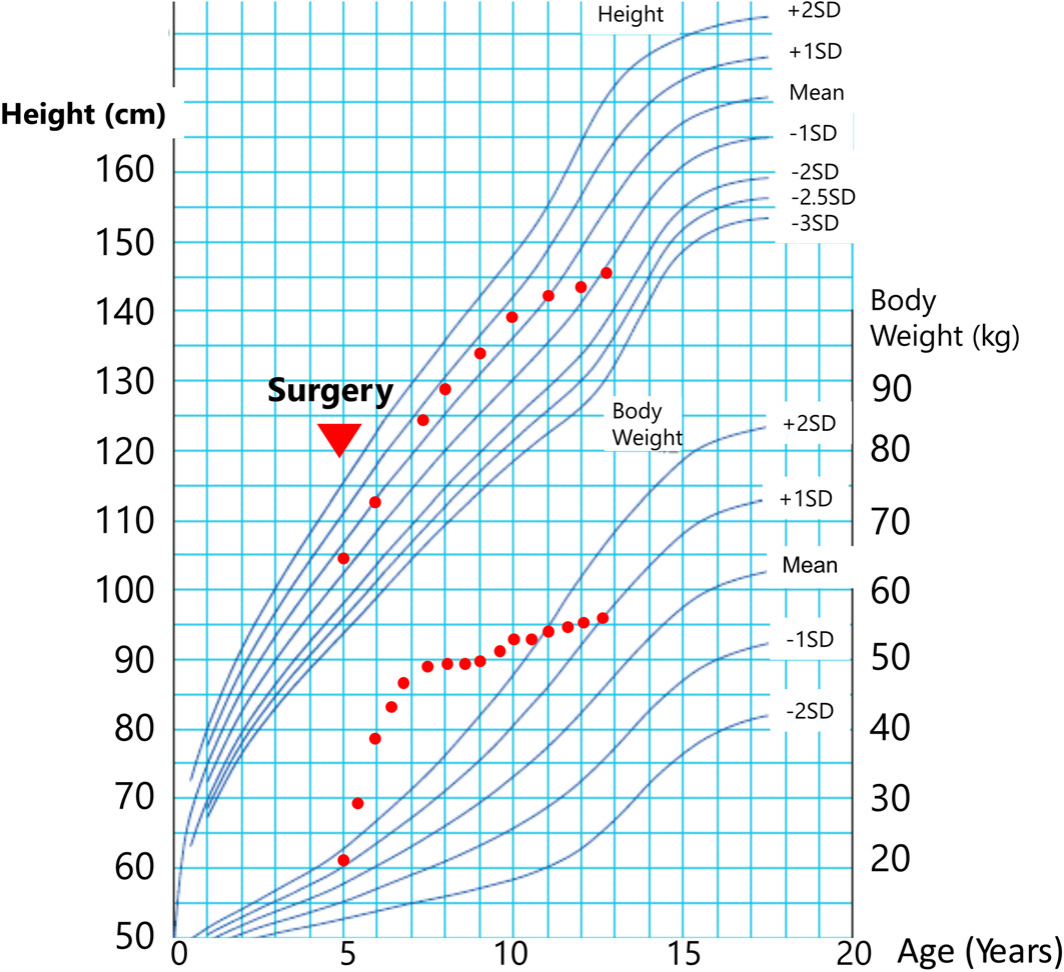
Growth chart. Growth chart revealed a rapid increase in body weight following craniopharyngioma surgery. At the age of 5 years, body weight remained stable due to adherence to strict diet therapy. Height growth continued normally without GH replacement.

The patient was a 13-year-old Asian boy diagnosed with panhypopituitarism due to craniopharyngioma at the age of 5 years. He initially presented with headaches, nausea, and vomiting. Imaging revealed a tumor occupying the third ventricle (**Supplementary Figure 1A**), and a biopsy confirmed it as an adenomatoid craniopharyngioma. At diagnosis, there were no signs of respiratory dysfunction or hepatomegaly. A craniotomy was performed to remove the tumor (**Supplementary Figure 1B**), and radiotherapy was not administered. Following tumor resection, he developed a thermoregulatory disorder and significant weight gain, with his BMI-SDS increasing from 22 to 44. Despite having markedly decreased insulin-like growth factor 1 (IGF-1) levels at the age of 8 and 10 years (8 and 9 nmol/L; age- and sex-specific reference ranges: 72–292 and 99–423 nmol/L, respectively), his height growth remained normal. Consequently, he did not meet the medical criteria for GH therapy and therefore did not receive GH treatment. He was instead treated with cortisol and levothyroxine for his panhypopituitarism, and his obesity was managed with a restricted diet, which improved his BMI-SDS from 44 to 35 (**Figure 1**). However, at 11 years of age, he was found to have a palpable liver enlargement of 4 cm, which increased to 7 cm below the costal margin by age 13. He also experienced frequent dyspnea, leading to hospital admission.

## 3 Diagnostic assessment, treatment, and outcome

**Figure 2:**
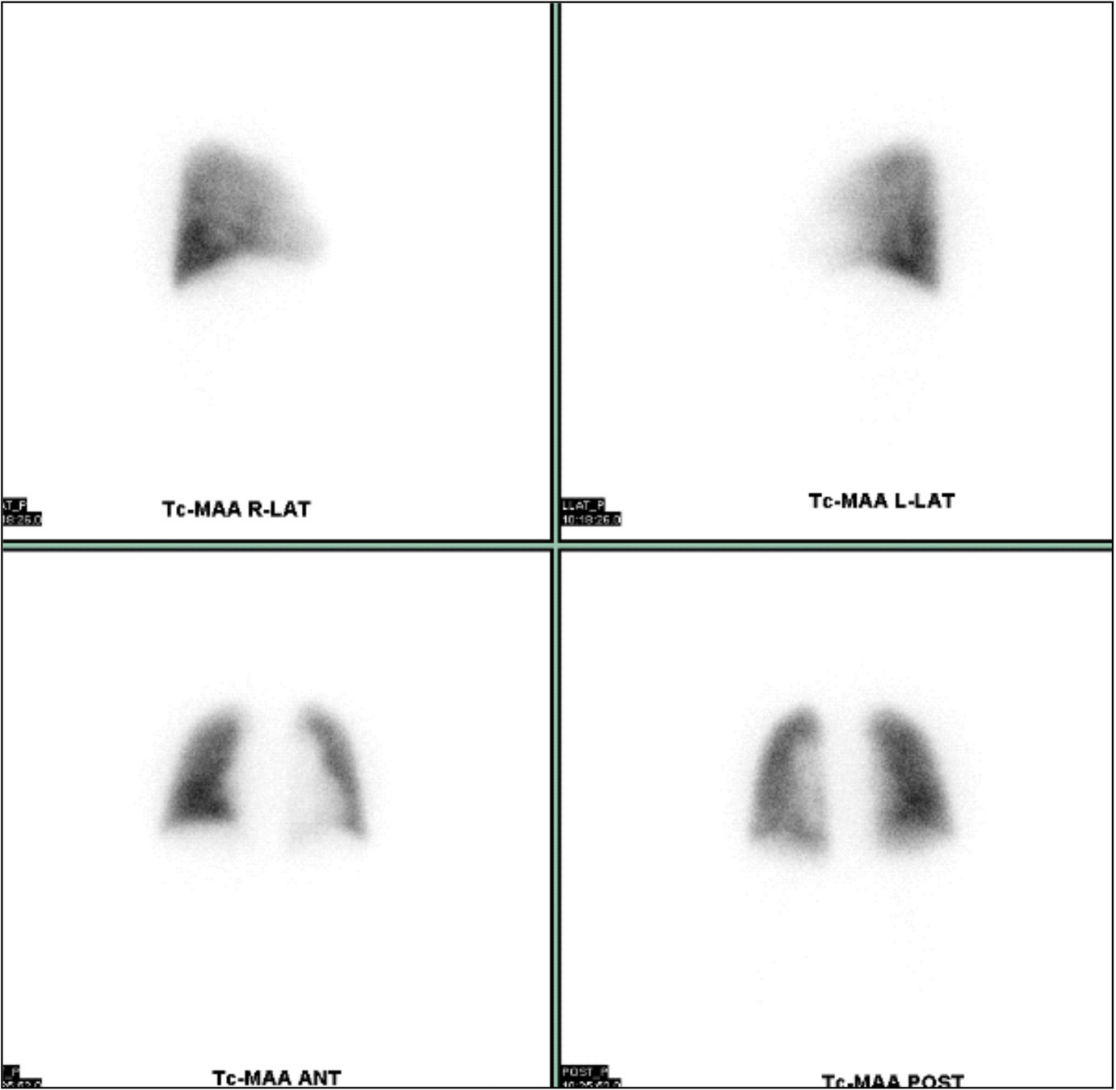
99mTc MAA pulmonary perfusion scintigraphy. 99mTc MAA pulmonary perfusion scintigraphy showed no lung perfusion defects but revealed a 10.5% increase in lung shunt.

Upon admission, his SpO_2_ level was 95%, and arterial blood gas analysis revealed a PaO_2_ of 78 mmHg and PaCO_2_ of 37 mmHg, indicating type 1 respiratory failure. While breathing room air, his alveolar–arterial oxygen difference was 29.2 mmHg. Contrast computed tomography scans did not show any pulmonary embolism or deep vein thrombosis. Additionally, 99mTc macroaggregated albumin (MAA) pulmonary perfusion scintigraphy revealed no lung perfusion defects but showed an increased lung shunt (10.5%; normal <5%) (**Figure 2**), suggesting intrapulmonary vascular dilatations that contributed to deoxygenation.

**Figure 3:**
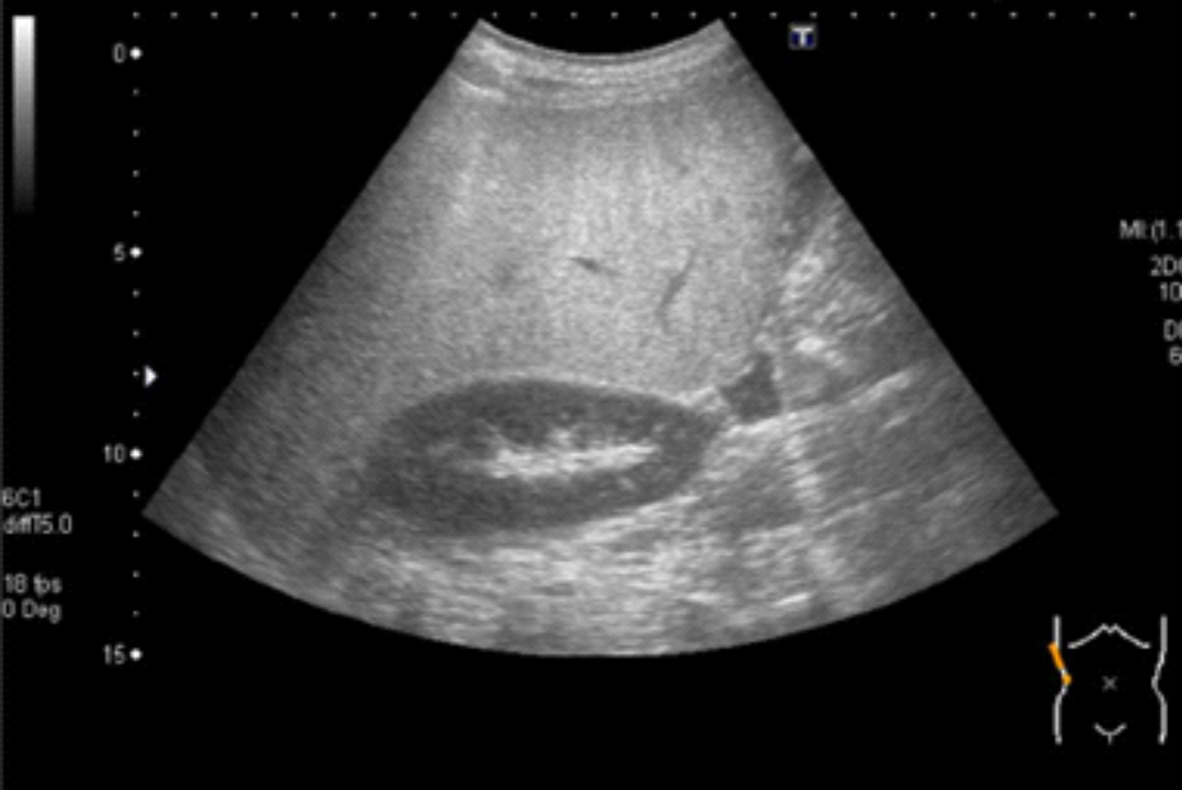
Abdominal ultrasound. Abdominal ultrasound revealed increased echogenicity in the liver compared with that in the kidney and spleen.

Abdominal ultrasound revealed increased echogenicity of the liver compared with the kidney and spleen (**Figure 3**). Magnetic resonance imaging showed liver inflammation, an irregular liver surface, and a splenorenal shunt. Magnetic resonance elastography revealed liver stiffness of 9.2 kPa (normal <2.0 kPa), and a liver biopsy indicated P-P bridging fibrosis and microvesicular fatty change, consistent with burnout nonalcoholic steatohepatitis. Consequently, the patient was diagnosed with HPS. In addition to a 1700 kcal diet therapy, home oxygen therapy was provided to alleviate his respiratory symptoms. The patient continues to receive treatment with branched-chain amino acids, a poorly absorbed antibiotic, and home oxygen therapy, and is regularly monitored during his visits to our hospital.

## 4 Discussion

HPS is a serious complication in individuals with liver disease, characterized by pulmonary vascular dilation and arterial hypoxemia due to increased blood flow in the lungs. Treatment generally focuses on addressing the underlying liver disease (e.g., liver transplantation) and managing respiratory symptoms with oxygen therapy or medications to improve pulmonary function (10). Our case implies that GH deficiency can potentially contribute to disease progression in pediatric patients with hypothalamic obesity and NAFLD. Despite efforts to control body weight, disease progression was observed, suggesting that weight control alone is insufficient. Typically, GH replacement therapy is reserved for children with growth retardation; however, some children with GH deficiency exhibit normal growth rates (11) and do not receive GH therapy. The retention of normal growth in these patients may be attributed to increased insulin and prolactin levels resulting from obesity (12). These children also develop several metabolic disorders, which GH replacement may improve, as demonstrated in an intervention study (9). Additionally, GH plays a role in regulating lipid metabolism in the liver (13). NAFLD is prevalent among adult patients with GH deficiency (14), and GH replacement therapy has been reported to improve liver conditions in these patients (15). This therapy may be also beneficial for children with craniopharyngioma and GH deficiency who develop hypothalamic obesity and HPS. However, GH replacement therapy is typically not indicated for children with GH deficiency who maintain a normal growth rate. Given that pharmacological interventions for children are controversial and not well-established, lifestyle modification remains the first-line treatment for NAFLD in this age group (16). Nevertheless, our case demonstrated that lifestyle modification with BMI-SDS reduction alone was insufficient. A review of the relationship between brain tumors and metabolic disorders indicated that NAFLD is caused not only by obesity but also by GH deficiency (7). GH reduces lipogenesis in hepatocytes, leading to hepatic steatosis independent of IGF-1 action (13, 17). Prior to our case, weight reduction in children with GH deficiency and fatty liver was not significantly observed (9), and poor weight control as the primary cause of liver disease progression rather than GH deficiency was not confirmed. Our case underscores that GH deficiency could be a potential contributor to NAFLD and HPS. For children with hypothalamic obesity and GH deficiency resulting from postoperative brain tumor surgery, GH replacement therapy may be necessary to prevent NAFLD and HPS, even in the presence of a normal growth curve. This case may encourage other clinical researchers to conduct more studies to expand the indications for GH treatment in children with craniopharyngioma and GH deficiency who maintain a normal growth rate but develop hypothalamic obesity.

## Data Availability

The raw data supporting the conclusions of this article will be made available by the authors, without undue reservation.

## Supplementary material

Supplementary Figure 1 Pre- and postoperative MRI imaging. **(A)** Preoperative sagittal T1-weighted brain MRI revealed a craniopharyngioma occupying the third ventricle. **(B)** Postoperative sagittal T1-weighted brain MRI revealed hypothalamic injury.
