# Supplementary figures and images for: Case report: Pediatric hepatopulmonary syndrome despite strict weight control after craniopharyngioma surgery
Source: Front Endocrinol (Lausanne). 2024 Oct 30;15:1459451. doi: 10.3389/fendo.2024.1459451 (PMC11557431; doi:10.3389/fendo.2024.1459451)

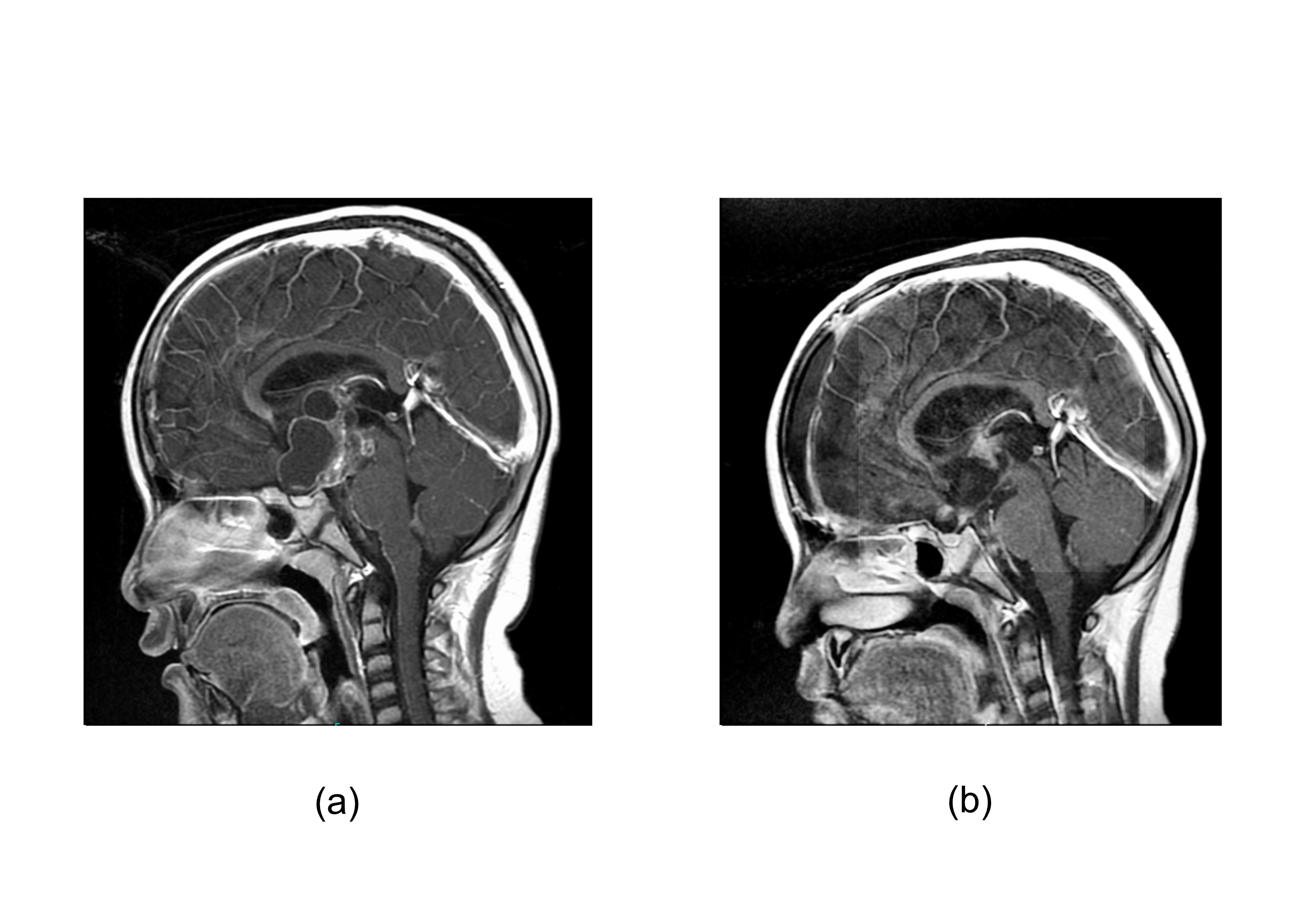

Supplement: Supplementary Figure 1 — Pre- and postoperative MRI imaging. (A) Preoperative sagittal T1-weighted brain MRI revealed a craniopharyngioma occupying the third ventricle. (B) Postoperative sagittal T1-weighted brain MRI revealed hypothalamic injury. [file Image1.jpeg]
